# Teacher trust in others and teacher commitment co-mediate the path from school leadership to students’ reading literacy in rural Chinese schools

**DOI:** 10.3389/fpsyg.2022.992003

**Published:** 2022-10-06

**Authors:** Haixue Zhu, Ling Li, Hui Li

**Affiliations:** ^1^Center for Education Policy, Southwest University, Chongqing, China; ^2^College of Educational Science, Chuzhou University, Anhui, China; ^3^Macquarie School of Education, Macquarie University, Sydney, NSW, Australia; ^4^Institute of Early Childhood Education, Shanghai Normal University, Shanghai, China

**Keywords:** school leadership, teacher trust in others, teacher commitment, reading literacy, serial mediation model

## Abstract

This study aimed to understand how teacher trust in others (TTO) and teacher commitment (TC) co-mediate the path from school leadership (SL) to students’ reading literacy (RL). Altogether 1,223 Grade 8 students (female =647; male = 576, *M_age_* = 15.35, *SD* = 1.28) and their 34 principals from 34 secondary schools in rural western China were sampled and matched. All the students completed the Program for International Student Assessment (PISA 2008) reading tests, and their principals completed the *Leading and Teaching in Schools Survey Scale* online which the variables of SL, TTO, and TC were evaluated. In addition, the “many to many” step was employed to match principals’ data with the students’ data by STATA analysis. The results indicated that: (1) there were direct and indirect effects of SL on student RL in the mediation model; (2) the serial mediation of TTO and TC was significant between SL and RL. This finding implies that enhancing TTO and TC in rural schools will help improve student’s RL.

## Introduction

Teacher emotion is one of the important keys to the improvement of student achievement in schools. According to the Ontario Leadership Framework (OLF; Leithwood, 2012), teachers’ “emotions path” includes TC, teacher trust in others (TTO), and teacher-collective efficacy. The existing literature has confirmed the important role of teachers’ “emotions path” in mediating between school leadership (SL) and student achievement in North American schools (Leithwood et al., 2010, 2019a,b; Handford and Leithwood, 2019). Recently, Zhu et al. (2020) have verified this mediating role in rural Chinese schools. However, the mediating effect of “emotions path” between SL and student achievement was negative, indicating that there might be some unidentified factors moderating or even mediating this mediating effect. From the previous study, every path of the OLF contained multiple variables and each variable played a different role in the path. So, it was important to understand how the variables interacting in the paths between SL and student achievement (Zhu et al., 2020). For instance, TTO and TC were highly correlated and might impact their emotions (Lau and Rowlinson, 2009; Buvik and Tvedt, 2016; Ghazinejad et al., 2018). And in particular, the variables of TTO and TC had been also found that had an indirect effect between SL and student achievement by Emotions path (Sun and Leithwood, 2015; Handford and Leithwood, 2019; Leithwood et al., 2019b). Therefore, TTO and TC might play mediating or moderating roles in the path from SL to student achievement. To verify their roles, this study proposed a serial mediation model and tested it with the data from 1,223 students and 34 secondary school principals in rural western China.

### School leadership on reading literacy

School leadership can mobilize and work with others to achieve common goals and has two core functions: setting direction and influencing others (Leithwood and Riehl, 2005). The relationship between SL and student achievement has been extensively explored by leadership scholars, who aimed to unveil the impacting mechanism of SL on student achievement. Two research directions have emerged from the existing studies. The first direction was exploring the direct effect of SL on student achievement. For instance, the original Claim 1 made by Leithwood et al. (2008) indicated that school leaders had a direct effect on student achievement: “school leadership is second only to classroom teaching as an influence on pupil learning.” And this direct effect has also been proved by many studies (Short and Spencer, 1989; Lambert, 1998; Nettles, 2005; Silva et al., 2011; Day et al., 2016; Hitt and Tucker, 2016; Huang and Zhao, 2017). In particular, Nettles (2005) found that SL could directly influence students’ reading achievement, labeled as instructional leadership. In the Chinese context, Huang and Zhao (2017) also found SL positively affected students’ reading literacy (RL) examined by the PISA 2012. In addition, a recent study in rural China (Zhu et al., 2020; Li et al., 2022) also found a direct effect of SL on student achievement.

The second direction was examining the indirect effect between SL and student reading achievement. Hallinger et al. (1996) had surveyed 36 school superintendents and an indirect effect of instructional leadership on student reading achievement. Later, Mitchell and Tarter (2016) found that the relationship between SL and reading achievement was mediated by school academic optimism climate. In addition, Leithwood et al. (2019b) examined the State of Texas Assessments of Academic Readiness program results and found that ‘rational path’ had a significant indirect effect. And SL impacted the reading achievement through the ‘rational’ and ‘emotions paths’. Recently, Zhu et al. (2020) verified this finding with the data from rural schools in western China and found that SL had a significant indirect effect on RL through emotions and organizational path. However, the “emotions path” mediating effect was negative, indicating that some unidentified factors such as TTO and TC should be involved in the model. This study will explore their possible mediating effects between the path from SL to RL to fill this gap.

### The role of teacher trust in others

Trust is necessary for teachers to actively and continuously participate in school improvement (Bryk and Schneider, 2002). Teacher Trust in Others includes the trust of the principals, colleagues, parents, and students (Leithwood et al., 2017). The existing studies have found that TTO was highly correlated with school effectiveness, school atmosphere and was also influenced by school environment and SL (Tarter et al., 1995; Hoy et al., 1996; Goddard et al., 2001; Leithwood et al., 2010; Hallam et al., 2015). This might be because the tone of the school atmosphere, especially the atmosphere of trust, was established by school leaders (Bryk et al., 2010). With the trust in school leaders, teachers could collaborate around a unified school goal and plan to drive school improvement collaboratively (Bryk et al., 2010). Therefore, TTO in principals might directly or indirectly impact student achievement (Chughtai and Buckley, 2009; Supovitz et al., 2010; Notman and Henry, 2011; Salfi, 2011; Sebastian and Allensworth, 2012; Forsyth and Adams, 2014; Tschannen-Moran, 2014; Zeinabadi, 2014; Heather et al., 2015). In particular, Heather et al. (2015) found that TTO not only had a direct effect and an indirect effect through school atmosphere factors (such as teacher’s professionalism, academic atmosphere, and learning community).

Accordingly, some studies have explored the mediating role of TTO in the relationship between SL and student achievement. For instance, Heather et al. (2015) found that TTO played a mediating role between SL and student achievement and was significantly correlated with academic atmosphere, teachers’ collective effectiveness, and teachers’ professionalism. In addition, TTO was one of the key variables in the “emotions path” proposed by Leithwood’s team, and its mediating effect has been verified by the team (Sun and Leithwood, 2015; Handford and Leithwood, 2019; Leithwood et al., 2019b). Furthermore, this finding was verified with the data from rural Chinese schools (Zhu et al., 2020). Therefore, this study will explore its mediating role between SL and student achievement in rural Chinese schools.

### The role of teacher commitment

As one of the key variables in the “emotions path” proposed by the Ontario Leadership Framework (Leithwood, 2012), TC has four dimensions: commitment to teaching, to students, to organization, to change. On the one hand, TC was found highly associated with principals’ behaviors and leadership in a school environment (Hoy et al., 1990; Hallinger, 2003; Nguni et al., 2006; Fancera and Bliss, 2011; Lambersky, 2016; Qadach et al., 2019; Cansoy et al., 2020). For instance, many studies (Sabir et al., 2011; Fasola et al., 2013) have found a significant correlation between SL and TC. Recently, Cansoy et al. (2020) found that instructional leadership had a direct impact on TC. Some recent studies also confirmed the close relationship between SL and TC (Li et al., 2018; Zhu et al., 2020). On the other hand, the existing literature has confirmed a significant direct relationship between TC and student achievement (Rosenholtz, 1989; Hatton, 1997; Harvey et al., 1998; Housego, 1999; Ingersoll, 2001; Strahan et al., 2001; Caprara et al., 2006; Johnson et al., 2012; Pranita, 2018). For example, Rosenholtz (1989) found that TC could significantly predict student achievement in reading and math. All these findings jointly indicated that TC might mediate SL and student achievement. Therefore, this study would test this mediating effect with data from rural Chinese schools.

### The context of this study

A recent study on Chinese schools found that the “emotions path” was the important mediator in the path from SL and student achievement, and the key variables of “emotions path” such as TTO or TC played different roles (Zhu et al., 2020). However, the inter-relationships between TTO and TC have not been explored. For instance, TTO was found significantly correlated to TC and affected team performance, directly or indirectly (Jones and George, 1998; Brower et al., 2000; Dirks and Ferrin, 2001; Burke et al., 2007; Yang and Mossholder, 2009; Rachid et al., 2011). In particular, TTO was found a necessary factor to maintain teachers’ commitment to school improvement, participation in collaboration, and professional learning (Tarter et al., 1995; Bryk and Schneider, 2002, 2003; Louis, 2007; Tschannen-Moran, 2014; Liu et al., 2016), and could enhance TC (Ghazinejad et al., 2018). In addition, as for the composition of TTO mentioned above, TTO included teachers’ trust to principals, colleagues, parents and students. As for teacher trust in principals, studies had shown a significant correlation with TC (Hoy and Tschannen-Moran, 1999; Aryee and Chen, 2006; Leithwood et al., 2010; Thoonen et al., 2012; Sleegers et al., 2014). Similarly, teacher trust in their colleagues was to a large extent related to the central mission of the school. The higher the degree of trust among teachers, the more willing they were to collaborate with their colleagues, and the higher their commitment to students would be (Tschannen-Moran, 2009). Studies had also pointed out that TTO was a necessary factor in maintaining teachers’ commitment to school changing and professional learning (Tarter et al., 1995; Bryk and Schneider, 2002, 2003; Tschannen-Moran, 2004; Louis, 2007). This meant there was a close relationship between TTO and TC, and TTO can significantly predict TC.

**Figure 1:**
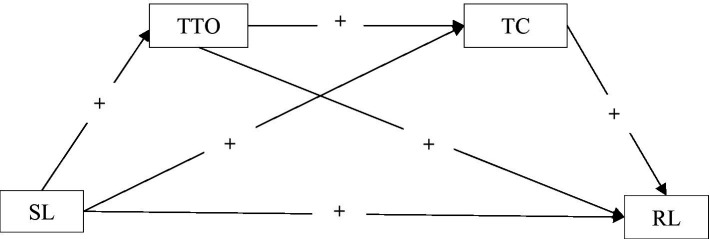
Proposed serial mediation model linking school leadership to student reading literacy in rural China. SL, School leadership; TTO, Teacher trust in others; TC, Teacher commitment; RL, Reading literacy.

Therefore, we hypothesized that TTO and TC might be the serial mediators in the path from SL to student achievement and proposed a serial mediation model (see Figure 1) to examine in this study. In particular, this study would examine the following hypotheses:

*H1*: School leadership have a direct impact on students’ RL.

*H2*: Teacher trust in others mediate the relationship between SL and students’ RL.

*H3*: Teacher commitment mediate the effect of SL on students’ RL.

*H4*: Teacher trust in others and TC play the serial mediation model fit the relationship between SL and RL.

## Materials and methods

### Participants

Whole group sampling was adopted to recruit all the Grade 8 students (aged around 15 years) from the 106 rural secondary schools in county D in western China. All the participating students were briefed about the survey by a trained graduate researcher and consented to participate in this study. And as the same time, their principals and class teachers were also invited to complete an online survey in December 2018 to provide information on SL, TC, and collaborative culture on line to explore the principal SL provide important support influence on student achievement. The “many to many” command were conducted with Stata software using school names as the matching identifier to match the school principals with their students. In total, the data from 34 school principals were matched with their 1,223 students (female = 647, 52.9%; Male = 576, 47.1%; *M_age_* = 15.35 years, *SD* = 1.28).

### Measures

#### Reading literacy

Student’s RL was assessed using the publicly released Chinese version of the PISA exam. An internationally standardized academic achievement test organized by the Organization for Economic Co-operation and Development (OECD), PISA aims to evaluate the academic performance of 15-year-olds in the subjects of reading. Its Chinese version was first applied to the 15-year-olds in Shanghai in 2009 (Sellar and Lingard, 2013), and later on, was released online for public use. In this study, the official Chinese versions of the Reading (2015) tests were employed to evaluate the students’ RL. The raw score for each student was converted to a standardized score (*z* score) in the statistical analysis in this study. RL test was consisting of 22 questions on seven mathematical topics which had been covering the three ability dimensions of “search and retrieval,” “integration and interpretation” and “reflection and evaluation.”

#### School leadership

The *Leading and Teaching in Schools Survey Scale* (Leithwood, 2017) was translated into Chinese and adopted to measure SL. This questionnaire has four constructs and 20 items. And a previous study has confirmed that the Chinese version was reliable and validated (Zhu et al., 2020; Li et al., 2022). The participants were asked to rank the factual statements such as: “Encourage collaborative work among staff,” “Work effectively with your teachers following classroom observation, to help them improve their instruction.” Reliability analysis showed the value of Cronbach α was 0.80, and the half-scale reliability was 0.77 which might be reliable used as the indicator of School Leadership in this study (Zhu et al., 2020).

#### Teacher commitment and teacher trust in others

In addition, this scale includes the measures of TC (5-item construct) and TTO (5-item construct; Leithwood, 2017). In TC scale, the participants were asked to rank the factual statements such as: “I volunteer to help my school colleagues when I think I can be useful to them,” “I am willing to “go the extra mile” to help my school achieve its goals for our students.” Reliability analysis on TC showed the value of Cronbach α was 0.77, and the half-scale reliability was 0.79 (Zhu et al., 2020). In TTO scale, the participants were asked to rank the factual statements such as: “Teachers can count on support from most students’ families.” “Teacher in my school trust our principal to provide the support we need to do our work well.” Reliability analysis on TTO showed the value of Cronbach α was 0.63, and the half-scale reliability was 0.77 (Zhu et al., 2020).

### Analytical strategy

First, SPSS 25.0 was adopted to explore SL, TTO, TC, RL, and control variables through descriptive statistics and correlation analysis. Second, the macro-program PROCESS 3.2 (Hayes, 2013) mediation analysis (by Model 6, 5,000 resamples, and 95% CI) was conducted to explore the serial mediating effects of TTO and TC between SL and students’ RL. Accordingly, we have tested whether the influence of SL on RL varies with a serial mediation model by TTO and TC.

## Results

### Preliminary analyses

The descriptive statistics and the intercorrelations among the studied variables for the total sample are presented in Table 1. SL was found significantly and positively correlated with TC (*r* = 0.473, *p* < 0.01), TTO (*r* = 0.460, *p* < 0.01) and RL (*r* = 0.065, *p* < 0.05). And TC was significantly and positively correlated with TTO (*r* = 0.802, *p* < 0.01), but not significantly with students’ RL a (*r* = −0.002, *p* > 0.05). TTO was significantly and negatively correlated with RL (*r* = − 0.059, *p* < 0.05). This correlation matrix tends to support the following PROCESS analysis to examine the hypothesized model.

**Table 1:** Descriptive statistics and pearson correlations among study variables.

	1	2	3	4
1. School leadership	–			
2. Teacher trust in others	0.460^**^	–		
3. Teacher commitment	0.473^**^	0.802^**^	–	
4. Reading literacy	0.065^*^	−0.059^*^	−0.002	–
*M*	4.179	4.172	4.016	456.568
SD	0.547	0.667	0.791	71.190

* *p* < 0.05 and

** *p* < 0.01 (two-tailed).

### Testing the serial mediation model

The PROCESS results are presented in Table 2, reflecting the relationship among SL, TTO, TC, and RL. First, SL had a significant direct effect on RL, *β* = 0.104, *p* < 0. 01. Second, there was an indirect effect of SL on RL through TTO and TC. In particular, SL could predict TTO (*β* = 0.460, *p* < 0. 001) and TC (*β* = 0.133, *p* < 0.001), and TTO could predict TC (*β* = 0.7413, *p* < 0.001) and RL (*β* = − 0.185, *p* < 0. 01). And TC could predict RL, *β* = 0.097, *p* < 0. 05.

**Table 2:** Path analysis results.

Paths	*β*
School leadership on Reading literacy	0.104^**^
School leadership on Teacher trust in others	0.460^***^
School leadership on Teacher commitment	0.133^***^
Teacher trust in others on Teacher commitment	0.741^***^
Teacher trust in others on Reading literacy	−0.185^**^
Teacher commitment to Reading literacy	0.097^*^

* *p* < 0.05,

** *p* < 0.01, and

*** *p* < 0.001 (two-tailed).

**Figure 2:**
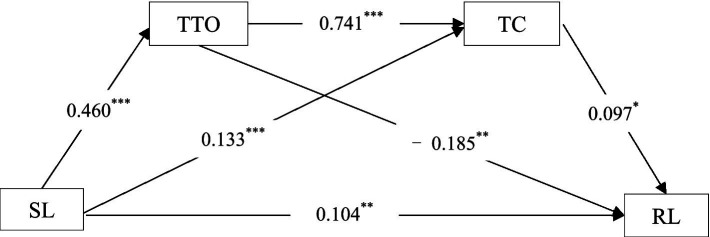
Examination of serial mediation: teacher trust in others and teacher commitment as serial mediators. SL, School leadership; TTO, Teacher trust in others; TC, Teacher commitment; RL, Reading literacy. *p < 0.05, **p < 0.01, ***p < 0.001 (two-tailed).

The best-fit modelling (Model 6) results indicated that: (1) the indirect effect of SL → TTO → RL was significant, *β* = −0.085, *SE* = 0.023, 95% CI = (−0.133, −0.041), not crossing zero; (2) the indirect effect of SL → TC → RL was also significant, *β* = 0.013, *SE* = 0.007, 95% CI = (0.001, 0.027), not crossing zero; (3) the indirect effect of SL → TTO → TC → RL was also significant, *β* = 0.033, *SE* = 0.016, 95% CI = (0.002, 0.065), not crossing zero. All these results jointly indicated that teacher trust and TC had a serial mediation effect on the relationship between SL and RL (see Figure 2; Table 3).

**Table 3:** Direct and indirect effects of school leadership on reading literacy.

Paths	*β*	*SE*	95% CI
School leadership → Reading literacy	0.104^**^	0.033	[0.040, 0.168]
School leadership → Teacher trust in others → Reading literacy	−0.085	0.023	[−0.133, −0.041]
School leadership → Teacher commitment → Reading literacy	0.013	0.007	[0.001, 0.027]
School leadership → Teacher trust in others → Teacher commitment → Reading literacy	0.033	0.016	[0.002, 0.065]

95% bias-corrected CIs reported as: [lower limit CI (LLCI), upper limit CI (ULCI)].

** *p* < 0.01 (two-tailed).

## Discussion

This study’s primary objective was to understand how TTO and TC jointly mediate the path from SL to RL in rural Chinese schools. The results have confirmed the serial mediating effects of TTO and TC. This section will discuss these findings and their implications for teacher development, SL, and improvement.

### Direct relationship between school leadership and reading literacy

This study found a significant direct impact of SL on students’ RL in rural Chinese schools. First, this might be attributed to the principals and their SL. This is because the rural schools in western China have so limited teaching resources that principals have to lead the managing and teaching simultaneously, and some even have to teach many classes (Zhu et al., 2020). In this specific circumstance, instructional leadership becomes the most prominent part of SL; accordingly, SL could directly affect student’s RL. Similar findings have been reported by Tan (2018) and Zhu et al. (2020), who found that Chinese principals tend to be instructional leaders in rural schools or disadvantaged contexts. In addition, as the leader of change, principals have to understand the school curriculum and pedagogy, plan for school improvement with teachers, and provide appropriate feedback to colleagues (Krug, 1992). All these instructional leadership activities will impact school learning environments, methods, and outcomes (Waters et al., 2003; Nettles, 2005; Day et al., 2016; Hitt and Tucker, 2016). Eventually, SL can influence students and their learning outcomes by setting high expectations and monitoring learning progress (Nettles et al., 2007; Kraft et al., 2015). Given the high dropout rates in rural schools of western china, principal’s every single measure or effort matters.

Second, the students, especially those in rural or disadvantaged contexts, are more sensitive to the principal’s instructional leadership. For example, Tan (2018) compared the impact of instructional leadership on different types of students and found that it could significantly improve disadvantaged students’ academic performance. In other non-Chinese contexts, SL was also found very effective in improving the academic performance of students with low SES background or in rural areas (Hallinger and Murphy, 1985; Andrews and Soder, 1987; Brewer, 1993; Kaplan et al., 2005; Robinson et al., 2008; Shatzer et al., 2014; Kraft et al., 2015; Dutta and Sahney, 2016). The rural schools in western Chinese are challenging for the principal, teachers, and students. In this challenging context, SL does have a direct impact on students’ RL.

### The serial mediation model of TTO and TC

This study has proposed and confirmed the hypothesized serial mediation model, indicating that TTO and TC mediate the path from SL to RL. This finding is a natural and further extension of the existing direction that TTO highly correlates with TC (Brower et al., 2000; Dirks and Ferrin, 2001; Burke et al., 2007; Lau and Rowlinson, 2009; Rachid et al., 2011; Buvik and Tvedt, 2016; Ghazinejad et al., 2018). First, TTO is necessary for them to participate actively and continuously in school improvement, especially in those challenging contexts (Bryk and Schneider, 2002; Zhang and Pang, 2016). On the basis of Piotr Sztompka Trust Theory, Trust was understood as a cultural rule which was an attribute of society as a whole. And the trust culture was considered as a product of history which seen as the result of collective, shared positive experiences of members of society over a long period of time. Trust behavior was built on three bases, namely the trust in others and the actor’s tendency to trust and trust culture (Sztompka, 1999). For the trust in others, in particular, TTO includes teacher trust in principals and teacher trust in colleagues (Tschannen-Moran and Hoy, 1998). Teacher trust in principals indicates that they believe that the principal will keep their promise and act for the school’s best interest. Teacher trust in their colleagues implies that they are confident that they can depend on each other, rely on each other, and be loyal to each other even in those challenging circumstances. In addition, teachers’ trust in the organization is also a key factor in maintaining their commitment to the school (Ferres et al., 2004). The higher trust in the school, the higher commitment to the school, and the more efforts to improve performance (McDonough, 2000). Therefore, TTO plays a significant mediating role in the path from SL to students’ RL.

Second, TTO is highly correlated with TC (Brower et al., 2000; Dirks and Ferrin, 2001; Burke et al., 2007; Lau and Rowlinson, 2009; Rachid et al., 2011; Buvik and Tvedt, 2016; Ghazinejad et al., 2018). This is because trust is the premise of commitment and has an important impact on commitment (Mohamed et al., 2012; Chen et al., 2015; Fard and Karimi, 2015; Martin and Siebert, 2016; Ghazinejad et al., 2018). Accordingly, TC could be strengthened if teachers trust their principals and colleagues (Ware and Kitsantas, 2007). This is why TTO and TC jointly play the roles of chain mediation in this study. In the rural schools of western China, even though principals directly impact students’ RL, they have to build an environment to nurture TTO and TC and establish a collaborative school culture. In such a challenging context, the rural teachers aspire for trust in others and other’s trust, which would enhance their faith and commitment level. With high levels of trust and commitment, they will be more willing to follow school leaders and join their efforts to improve teaching and learning. This will eventually pay off in students’ academic performance.

## Conclusion, limitations, and implications

This study has proposed and established a serial mediation model to demonstrate the joint mediating effects of TTO and TC in the relationship between SL and RL in rural western China. First, a significant direct effect of SL was found, indicating that principals could impact students’ RL directly in rural Chinese schools. Second, TTO and TC sequentially and jointly mediate the path from SL to students’ RL, indicating that teachers’ roles are important and influential.

However, this study has some major limitations. First, the number of students matched with the principals was small due to low return rates. This would have a certain influence on the conclusion. Second, the sample was limited to a county in rural China; thus, the findings could not reflect those schools in urban China. In the future, students from urban schools should be sampled to verify the findings of this study further.

Nevertheless, this study has some important implications for school management and teacher development. First, the finding that TTO and TC play a serial mediating role indicates that more attention should be paid to building a caring and collaborative school culture. In particular, the distributed leadership could be established in rural schools to build up a collaborative culture that values trust, commitment, and collaboration. Towards this ends, the professional learning community could be developed in these rural schools (Wang et al., 2017). With the help of this community, principals could fully maximize the subjectivity and potential of teachers and enhance their trust, commitment, and teaching. And more public and policy attention should be paid to the teachers’ emotions and interests in rural schools, who are suffering from the limited resources and disadvantaged environments (Liu, 2016). Second, SL training programs should be provided to those principals in rural schools and other disadvantaged areas. These in-service training will empower principals with the knowledge and skills to embrace the challenges and opportunities faced by their schools. Local educational authorities should be more aggressive, innovative, and proactive in developing and delivering these in-service training and supporting programs. These remedial measures to teachers and principals in rural schools will eventually help bridge the rural–urban gaps in student achievement and achieve educational equality and social justice.

## Data availability statement

The raw data supporting the conclusions of this article will be made available by the authors without undue reservation.

## Ethics statement

The studies involving human participants were reviewed and approved by the Ethical Committee, Faculty of Education, Southwest University. Written informed consent to participate in this study was provided by the participant’s legal guardian/next of kin.

## Author contributions

HZ conducted the study. LL and HL conducted the data analysis and drafted the manuscript. All authors contributed to the article and approved the submitted version.

## Funding

This paper is supported by Decision Making Laboratory for Western Education and Human Development, National Social Science and Humanity Foundation (18ZDA338), and 111 Program (B21036); National Educational Double First-Class Program at Southwest University, Innovation Research 2035 Pilot Plan of Southwest University (SWUPilotPlan004); Key projects of Outstanding Young Talents Support Program in Colleges and Universities (gxyqZD2020107) Chongqing Social Science and Humanity Foundation (2022YC028); and Youth Project of Philosophy and Social Science Foundation of Anhui Province (AHSKQ2020D176).

## Conflict of interest

The authors declare that the research was conducted in the absence of any commercial or financial relationships that could be construed as a potential conflict of interest.

## Publisher’s note

All claims expressed in this article are solely those of the authors and do not necessarily represent those of their affiliated organizations, or those of the publisher, the editors and the reviewers. Any product that may be evaluated in this article, or claim that may be made by its manufacturer, is not guaranteed or endorsed by the publisher.
